# RP-HPLC Separation and ^1^H NMR Identification of a Yellow Fluorescent Compound—Riboflavin (Vitamin B_2_)—Produced by the Yeast *Hyphopichia wangnamkhiaoensis*

**DOI:** 10.3390/biom13091423

**Published:** 2023-09-20

**Authors:** Raziel Arturo Jiménez-Nava, Luis Gerardo Zepeda-Vallejo, Fortunata Santoyo-Tepole, Griselda Ma. Chávez-Camarillo, Eliseo Cristiani-Urbina

**Affiliations:** 1 Departamento de Ingeniería Bioquímica, Instituto Politécnico Nacional, Escuela Nacional de Ciencias Biológicas, Avenida Wilfrido Massieu s/n, Unidad Profesional Adolfo López Mateos, Ciudad de Mexico 07738, Mexico; 2 Departamento de Microbiología, Instituto Politécnico Nacional, Escuela Nacional de Ciencias Biológicas, Prolongación de Carpio y Plan de Ayala s/n, Colonia Santo Tomás, Ciudad de Mexico 11340, Mexico; 3 Departamento de Química Orgánica, Instituto Politécnico Nacional, Escuela Nacional de Ciencias Biológicas, Prolongación de Carpio y Plan de Ayala s/n, Colonia Santo Tomás, Ciudad de Mexico 11340, Mexico; 4 Departamento de Investigación, Instituto Politécnico Nacional, Escuela Nacional de Ciencias Biológicas, Prolongación de Carpio y Plan de Ayala s/n, Colonia Santo Tomás, Ciudad de Mexico 11340, Mexico

**Keywords:** bio-separation, fluorescent compound, *Hyphopichia wangnamkhiaoensis*, RP-HPLC-DAD, ^1^H NMR, riboflavin, yeast

## Abstract

The yeast *Hyphopichia wangnamkhiaoensis* excretes a brilliant yellow fluorescent compound into its growth culture. In this study, we isolated and identified this compound using reverse-phase high-performance liquid chromatography-diode array detector (RP-HPLC-DAD) as well as ^1^H NMR and UV–Vis spectroscopy. Two of the three RP-HPLC-DAD methods used successfully separated the fluorescent compound and involved (1) a double separation step with isocratic flow elution, first on a C18 column and later on a cyano column, and (2) a separation with a linear gradient elution on a phenyl column. The wavelengths of maximum absorption of the fluorescent compound-containing HPLC fractions (~224, 268, 372, and 446 nm) are in good agreement with those exhibited by flavins. The ^1^H NMR spectra revealed methyl (*δ* 2.30 and 2.40) and aromatic proton (*δ* 7.79 and 7.77) signals of riboflavin. The ^1^H NMR spectra of the samples spiked with riboflavin confirmed that the brilliant yellow fluorescent compound is riboflavin. The maximum excitation and emission wavelengths of the fluorescent compound were 448 and 528 nm, respectively, which are identical to those of riboflavin.

## 1. Introduction

Fluorescent compounds, also known as fluorochromes or fluorophores, are widely used as markers in microscopy of biological samples, histology, molecular biology, immunology, material sciences, and chemistry. Several fluorochromes have also been used as drugs [1,2,3,4]. Fluorochromes are classified as intrinsic or extrinsic based on their luminescence principle and synthetic or organic (natural) based on their chemical origin [5]. Synthetic fluorochromes are the most commonly used fluorochromes owing to their higher stability in reaction to physicochemical changes. Notably, the vitamins riboflavin (B_2_), ergocalciferol (D_2_), phytomenadione (K_1_), and pyridoxine (B_6_); the amino acids phenylalanine, tyrosine, and tryptophan; and the vitamin precursors ergosterol and β-carotene are some important organic fluorochromes for human health [3,5,6].

Several organic fluorochromes are produced industrially by microorganisms [7,8,9,10] in high yields and with high specificity. Yeasts have attracted significant attention in recent years for their ability to biosynthesize fluorochromes. Yeasts are metabolically versatile, low maintenance, easy to cultivate, safe, and have a high specific growth rate and short duplication time [11,12]. Some of the fluorochromes produced by yeasts are as follows: (1) ergosterol, a precursor of vitamin D_2_, is produced by *Cystofilobasidium capitatum*, *Rhodotorula glutinis*, and *Sporobolomyces roseus*; (2) β-carotene, a precursor of vitamin A, is produced by the same yeast species mentioned previously (*C. capitatum*, *R. glutinis*, and *S. roseus*) [9,10,13,14,15], and (3) riboflavin, an essential vitamin for human nutrition, is produced by some yeast species such as *Candida famata* and *Meyerozyma guilliermoindii* (formerly known as *Candida* or *Pichia guilliermondii*) [16,17,18,19,20].

Nowadays, high-performance liquid chromatography (HPLC) is one of the most reliable analytical techniques for separating, quantifying, and identifying fluorochromes [21,22,23]. Likewise, nuclear magnetic resonance spectroscopy and mass spectrometry are powerful analytical tools that have been used to quantify known fluorochromes, as well as to elucidate the chemical identity, structure, and properties of fluorochromes [24,25,26].

*Hyphopichia wangnamkhiaoensis*, formerly known as *Candida wangnamkhiaoensis* and *Wickerhamia* sp. X-Fep, is a dimorphic yeast species capable of producing high levels of extracellular α-amylase [12,27,28,29] and oleic acid [30]. During the course of our investigation into the above-mentioned biosynthesis, we observed that the cultures of *H. wangnamkhiaoensis* turned yellow during the production of α-amylase and oleic acid, which was attributed to a yellow fluorescent compound. However, this compound has not yet been characterized in detail. Therefore, in this study, we isolated the bright yellow fluorescent compound produced by *H. wangnamkhiaoensis* using reverse-phase high-performance liquid chromatography (HPLC) and characterized it using nuclear magnetic resonance (NMR) and UV–visible and fluorescence spectroscopy. To the best of our knowledge, this is the first report of the isolation and structural characterization of a fluorescent compound produced by *H. wangnamkhiaoensis*.

## 2. Materials and Methods

### 2.1. Reagents

HPLC-grade methanol, water, acetonitrile, and other chemical reagents used in the culture medium were purchased from JT Baker (Avantor Performance Materials, Inc., Xalostoc, Estado de México, Mexico). Analytical standards of deuterated water (deuterium oxide) (D_2_O), biotin, and riboflavin were purchased from Sigma-Aldrich (Sigma-Aldrich, Co., Santa Clara, CA, USA).

### 2.2. Batch Cultivation of H. wangnamkhiaoensis

The *H. wangnamkhiaoensis* yeast strain was obtained from the Industrial Microbiology Laboratory Culture Collection of the National School of Biological Sciences, National Polytechnic Institute (ENCB-IPN), Mexico City, Mexico.

Modified Castañeda-Agulló’s [31] culture medium (10 g/L glucose, 4.85 g/L (NH_4_)_2_SO_4_, 0.625 g/L dibasic ammonium citrate, 1.0114 g/L KH_2_PO_4_, 0.275 g/L MgSO_4_·7H_2_O, 0.375 g/L Na_2_CO_3_, 0.250 g/L NaCl, and 0.02 mg/L biotin) was used for yeast cultivation.

The yeast was batch-cultivated in a bubble column pneumatic bioreactor for 30 h at 28 ± 2 °C, with a sterile air supply of 1.11 vvm. After incubation, the yeast cells were separated through centrifugation (5000 rpm, 10 min), and the supernatant was sterilized with microfiltration using mixed cellulose esters Millipore^®^ (Merck KGaA, Darmstadt, Germany) membranes of 0.22 μm pore size.

### 2.3. Concentration, Extraction, and Separation of the Fluorescent Compound Produced by H. wangnamkhiaoensis

The membrane-sterilized supernatant (400 mL) was lyophilized to concentrate the fluorescent compound and remove all the water. Subsequently, the lyophilized powder was leached out with 100 mL of HPLC-grade methanol and vortexed for 5 min at ambient temperature (25 ± 1 °C). The resulting mixture was separated through centrifugation (5000 rpm, 10 min). The methanolic extract was collected, and the methanol-insoluble phase was discarded. The methanolic extract was then subjected to HPLC.

Reverse-phase HPLC (RP-HPLC) analysis of the methanolic extract was performed using two isocratic elution methods (Methods 1 and 2) and one linear gradient elution method (Method 3), which are described below:

Method 1: RP-HPLC analysis was performed in an Agilent 1260 Infinity series system (Agilent Technologies, Inc., Santa Clara, CA, USA) equipped with a Zorbax SB-C18 column (250 mm × 4.6 mm, 5 μm particle size) and a diode array detector (DAD). The isocratic mobile phase was 20:80 (*v*/*v*) acetonitrile and water at a flow rate of 1 mL/min. Chromatograms were obtained at 280 nm because of the aromatic nature of several known fluorochromes and at 440 nm because a preliminary assay showed that the fluorescent compound-containing supernatant has the highest absorption in the visible region at this wavelength.

The fraction with the longest retention time on the HPLC column, a well-defined absorbance peak with a small width, and/or a high absorption at both 280 and 440 nm was then selected and dried in an oven at 40 °C. One portion of this fraction was used as a feedstock for Method 2, and another portion of the fraction was analyzed spectroscopically.

Method 2: The previously selected fraction from Method 1 was subjected to another analytical RP-HPLC separation using an Agilent 1260 Infinity series system equipped with a cyano (CN^−^) column (150 mm × 4.6 mm, 5 μm particle size Zorbax SB-CN) connected to a DAD. UV–Vis signals were collected at 280 and 440 nm. The isocratic mobile phase was 10:90 (*v*/*v*) acetonitrile and formic acid 0.1% (*v*/*v*) in water at a flow rate of 1 mL/min. The fraction with the longest retention time on the HPLC column, a narrow absorbance peak, and/or a high absorption at both 280 and 440 nm was selected and dried in an oven at 40 °C for further spectroscopic analysis and characterization.

Method 3: A modified version of the method reported by Odanaka et al. [23] was used. Briefly, 50 mL of the methanolic extract was evaporated to dryness at 35 ± 1 °C, and the resulting powder was dissolved in 10 mL of HPLC-grade water. Next, 3 mL of the resulting solution was loaded in triplicate onto C18 Alltech^®^ Maxi-Clean^TM^ solid phase extraction cartridges (Thermo Fisher Scientific, Inc., Waltham, MA, USA). The cartridges were eluted under vacuum conditions with 1.5 mL of methanol containing 0.002% (*v*/*v*) HCl. The eluate was collected and processed by analytical RP-HPLC using an Agilent 1260 Infinity series system equipped with a DAD (UV–Vis signals were traced at 280 and 440 nm) and a phenyl column (150 mm × 4.6 mm, 5 μm particle size, Zorbax SB-Phenyl (Agilent Technologies, Inc., Santa Clara, CA, USA)). The mobile phase was a mixture of acetonitrile and water containing 0.05% (*v*/*v*) phosphoric acid at a flow rate of 0.5 mL/min. The column was eluted using a linear gradient from 10 to 100% acetonitrile applied for 15 min. The fraction that exhibited the longest retention time on the HPLC column, a narrow absorbance peak, and/or a high absorption at both 280 and 440 nm was selected and dried in an oven at 40 °C for further spectroscopic analysis and characterization.

### 2.4. UV–Vis Spectroscopy Analysis of the Selected Fractions from RP-HPLC-DAD Analyses

The UV–Vis absorption spectra of the fractions selected from the three different RP-HPLC methods were obtained with the information provided by DAD and plotted using Agilent OpenLab CDS EZChrom A.0404 software (Agilent Technologies, Inc., Santa Clara, CA, USA).

### 2.5. The 1D ^1^H NMR Analysis

The one-dimensional (1D) ^1^H NMR spectra of the fractions selected from the three different RP-HPLC methods and a riboflavin standard solution were measured at 499.85 MHz using a Varian NMR 500 system (Agilent Technologies, Inc., Santa Clara, CA, USA) operating at 11.7 T equipped with a 5 mm OneNMR probe (Agilent Technologies, Inc., Santa Clara, CA, USA) at 25 °C, using deuterium oxide (D_2_O) as solvent. The 1D ^1^H NMR spectra were recorded without spinning using the PRESAT pulse sequence to suppress the residual H_2_O signal. The acquisition parameters for the ^1^H NMR observations were: 32k data points (np), 8012.8 Hz spectral width (sw), 2.0047 s acquisition time (at), 3.0 s delay time (d1), and 256 scans (ns). The data were zero-filled to 64k data points before Fourier transformation (FT).

### 2.6. The ^1^H NMR Spectra Data Processing

The ^1^H NMR spectra were processed using MestReNova 14.2.0 software from Mestrelab Research S.L. (Santiago de Compostela, Coruña, Spain).

### 2.7. Spectrofluorometric Characterization of the Fractions Selected from RP-HPLC Analyses

The excitation and emission spectra of the fractions selected from RP-HPLC-DAD analyses were measured at 24 ± 1 °C using a SpectraMax M3 fluorometer (Molecular Devices LCC, San Jose, CA, USA). A sweep from 450 to 550 nm at an emission wavelength (λ_em_) of 525 nm was used for excitation measurements, and a sweep from 300 to 500 nm at an excitation wavelength (λ_ex_) of 450 nm was used for emission measurements. These maximum fluorescence excitation and emission wavelengths were previously optimized to enhance the selectivity and sensitivity of the method [32].

## 3. Results and Discussion

### 3.1. RP-HPLC-DAD Analysis of the Supernatant of the H. wangnamkhiaoensis Liquid Culture

**Figure 1:**
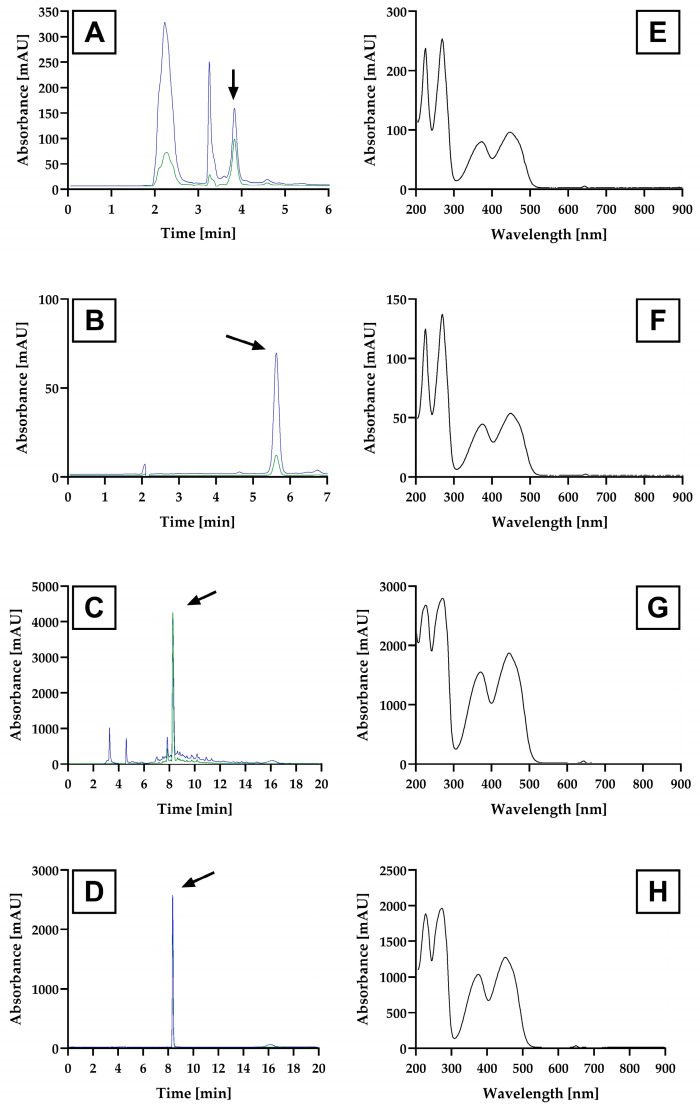
Reverse-phase high-performance liquid chromatography-diode array detector (RP-HPLC-DAD) chromatograms of (**A**) FCHw-M1, (**B**) FCHw-M2, (**C**) FCHw-M3, and (**D**) riboflavin standard (green plot: 280 nm signal; blue plot: 440 nm signal); UV–Vis spectra of (**E**) FCHw-M1, (**F**) FCHw-M2, (**G**) FCHw-M3, and (**H**) riboflavin standard. The arrow marks in (**A**–**D**) show the selected HPLC fractions whose UV–Vis spectra are displayed in (**E**–**H**).

The yellow fluorescent compound excreted into the culture supernatant of *H. wangnamkhiaoensis* was analyzed by three RP-HPLC-DAD methods. Several fractions were obtained from RP-HPLC-DAD Method 1 (Figure 1A). The fraction with the longest retention time (3.8 min), a high absorption at both 280 and 440 nm, and a narrow absorbance peak width (marked with an arrow in Figure 1A) was selected and named FCHw-M1. Similarly, major narrow peaks with retention times of 5.3 min (marked with an arrow in Figure 1B) and 8.2 min (marked with an arrow in Figure 1C) and exhibiting the highest absorption at both 280 and 440 nm were selected from RP-HPLC-DAD Methods 2 and 3 and named FCHw-M2 and FCHw-M3, respectively.

### 3.2. UV–Vis Characterization of the Fractions Selected from RP-HPLC-DAD Analyses

The UV–Vis absorption spectra of FCHw-M1, FCHw-M2, and FCHw-M3 are shown in Figure 1E–G. All UV–Vis spectra showed comparable profiles and four peaks with similar wavelengths of maximum absorption (λ_max_). FCHw-M1 and FCHw-M2 exhibited peaks with λ_max_ at 224, 268, 372, and 446 nm (Figure 1E,F), whereas FCHw-M3 showed peaks with similar λ_max_ at 225, 270, 370, and 446 nm (Figure 1G).

Flavins exhibit four characteristic absorption peaks at λ_max_ of approximately 220, 265, 375, and 445 nm [33,34], which are consistent with the spectra of the fluorescent compound produced by *H. wangnamkhiaoensis*. Furthermore, flavins are pale yellow, water-soluble, fluorescent organic compounds [34,35,36]; such characteristics are also exhibited by the yellow fluorescent compound produced by the yeast strain. Flavins such as riboflavin (RF) and its derivatives, flavin mononucleotide (FMN) and flavin adenine dinucleotide (FAD), are commonly found in all living organisms. These are the most biologically important flavins owing to their essential role in oxidation–reduction (redox) reactions involved in energy production, cellular antioxidant functions, and numerous metabolic pathways, all of which impact human health [34,35,37]. Flavins such as formylmethylflavin (FMF), carboxylmethylflavin (CMF), lumiflavin (LF), and lumichrome (LC) derived from the photolysis and oxidation of RF are found in milk, dairy products, and the culture supernatant of some flavinogenic microorganisms [33,34,38,39,40,41,42,43,44,45,46,47]. LC is biosynthesized by organisms of diverse origins such as the bacterium *Nocardia alba* [48], the ascidian *Halocynthia roretzi* [49], and the herbaceous plant *Galactites tomentosa* Moench [50].

The fluorescent compound excreted by *H. wangnamkhiaoensis* was successfully separated by the three RP-HPLC-DAD methods used. Similarly, flavins from foods, milk, dairy products, beverages [42,43,50,51,52,53,54,55,56,57,58], plant extracts [59], pharmaceutical products [46,60], culture supernatants of microorganisms [23,41,45], *Keroplatus* larvae [25] and the ascidian *H. roretzi* [49] have been previously separated using HPLC methods.

The UV–Vis spectra of most of the above-mentioned flavins were comparable to each other [33,38,40,42,43,44,46,61]. The λ_max_ of RF produced by *Bordetella pertussis* (225, 270, 370, and 450 nm) [23], dissolved in methanol (270–271, 344–358, and 440–450 nm) [61], dissolved in phosphate and borate buffers (362 and 440 nm in the visible region) [62], and separated from multivitamin tablets (222, 267, 369, and 445 nm) [60] and from pig liver (220, 267, 361, and 462 nm) [43] are nearly identical to those reported for CMF (223, 266, 376, and 445 nm) [63] but slightly different from those reported for LC (225, 258, 353, and 388 nm [41] and 218, 261, 355, and 382 nm [48]). The slight differences in the λ_max_ of RF could be attributed to the solvent effect and hydrogen bonding interactions [33,61].

The fluorescent compound was further characterized by ^1^H NMR to confirm whether it was a flavin.

### 3.3. The ^1^H NMR Characterization of the Fractions Selected from RP-HPLC-DAD Analysis

**Figure 2:**
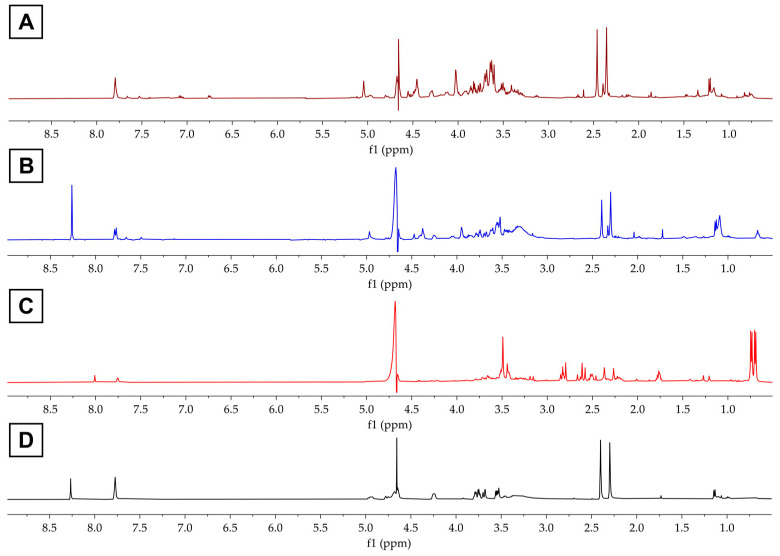
The ^1^H NMR (500 MHz, D_2_O) spectra of (**A**) FCHw-M1, (**B**) FCHw-M2, (**C**) FCHw-M3, and (**D**) riboflavin standard.

The ^1^H NMR spectrum of FCHw-M1 showed a large number of signals (Figure 2A), making it difficult to identify the signals corresponding to the fluorescent compound. These results suggest that RP-HPLC-DAD Method 1 is not suitable for separating the fluorescent compound from the culture supernatant of *H. wangnamkhiaoensis*. Hence, FCHw-M1 was no longer analyzed further.

In contrast, the ^1^H NMR spectrum of FCHw-M2 revealed two single signals at *δ* 2.30 and 2.40 ppm (Figure 2B), which correspond to methyl protons. The other group of signals corresponds to aromatic protons at *δ* 7.79 and 7.77 (Figure 2B). Similarly, the ^1^H NMR analysis of FCHw-M3 shows methyl proton signals at *δ* 2.27 and 2.37 (Figure 2C) and aromatic proton signals at *δ* 7.76 and 7.75. Moreover, the spectrum of FCHw-M3 exhibited signals of citrate (*δ* 2.58, 2.61, 2.80, and 2.83) present in the culture medium (Figure 2C), which were not observed for FCHw-M2 (Figure 2B).

The ^1^H NMR spectroscopy has been previously used to elucidate the structure of flavins, such as FMN [64,65,66,67,68,69,70], FAD [66,67,68,71], LF [72], LC [48,49,50,72], and RF [25,73,74]. The ^1^H NMR spectral characteristics in this study are consistent with those of RF in deuterated dimethyl sulfoxide (DMSO_d6_) reported by Malele et al. [73], where methyl proton signals at *δ* 2.36 and 2.45 and aromatic proton signals at *δ* 7.84 and 7.78 were observed. The ^1^H NMR spectrum of an RF reference standard dissolved in a deuterated trifluoroacetic acid/trifluoroacetic anhydride mixture (TFA_d_:TFAA) showed methyl group protons at *δ* 2.11 and 2.24 and aromatic proton signals at *δ* 7.63 and 7.44 [74]. Similarly, the ^1^H NMR spectrum of RF isolated from *K. testaceus* larvae and dissolved in deuterated water showed methyl protons at *δ* 2.5 and 2.6 and aromatic protons at *δ* 7.93 and 7.96 [25]. These chemical shift variations can be attributed to the solvent used to dissolve RF for ^1^H NMR spectral analysis. Furthermore, the chelating effect of the citrate ion present in FCHw-M3 can affect the NMR chemical shifts of RF and other chemical compounds [75,76].

The ^1^H NMR spectra of FMN [64,66,69,70,77], LC [48,49,50,72], and LF [72] are easily distinguishable from the RF spectrum due to their intrinsic structural differences. Compared to RF, FMN has a phosphate group, LC has an additional imino group, LF has an additional methyl group, and both LC and LF do not contain the d-ribityl side chain. The ^1^H NMR spectrum of the fluorescent compound produced by *H. wangnamkhiaoensis* is consistent with that of RF and substantially differs from the spectra of the other flavins [25,73,74]. Similarly, the UV–Vis spectra of the fluorescent compound in FCHw-M2 and FCHw-M3 are comparable to those of other flavins, particularly RF [23,33,53,60,61,62]. Therefore, the brilliant yellow fluorescent compound produced by *H. wangnamkhiaoensis* is confirmed to be RF.

Most yeasts can synthesize RF only to the extent of their own requirements; only a few yeasts overproduce and excrete RF into the fermentation broth [7,8,78,79,80,81,82,83]. *Candida*, *Schizosaccharomyces*, *Schwanniomyces*, *Meyerozyma*, *Pichia*, *Hyphopichia*, and *Debaryomyces* genera produce RF [7,80,84]. However, to the best of our knowledge, the production of RF by *H. wangnamkhiaoensis* has not been described thus far.

### 3.4. RP-HPLC-DAD, UV–Vis, and ^1^H NMR Analyses of Riboflavin Standard

To support our structural conclusion, we analyzed an RF reference standard (Sigma-Aldrich) via RP-HPLC-DAD Method 3 (Figure 1D), UV–Vis spectroscopy (Figure 1H), and ^1^H NMR (Figure 2D). A single peak at the retention time of 8.4 min (Figure 1D) was observed, which is similar to that obtained for FCH_W_-M3 (8.2 min). Furthermore, the UV–Vis spectrum of the RF standard revealed four peaks of maximum absorption at 225, 270, 370, and 446 nm (Figure 1H), which were also observed for FCHw-M2 and FCHw-M3.

The ^1^H NMR spectrum of the RF standard (Figure 2D) exhibited methyl proton signals at *δ* 2.30 and 2.40, d-ribityl proton signals (*δ* 3.50–4.50), and an aromatic proton signal (*δ* 7.77), which were also observed for FCH_W_-M2 and FCH_W_-M3. The chemical shift variations may be ascribed to changes in sample concentration and measurement temperature, as previously reported for the aromatic protons in flavins [66,67,68,71,85].

### 3.5. Spike-In ^1^H NMR Experiments

**Figure 3:**
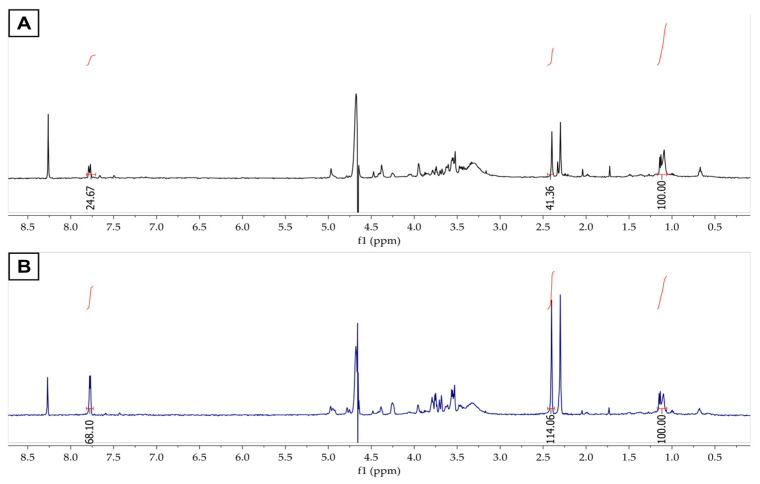
The ^1^H NMR spectra of (**A**) FCHw-M2 and (**B**) FCHw-M2 spiked with riboflavin.

To confirm the identity of the fluorescent compound, spike-in ^1^H NMR experiments with the RF standard were performed. The RF standard solution was added to the FCHw-M2 and FCHw-M3 ^1^H NMR samples, and the corresponding spectra were recorded. In the ^1^H NMR spectrum of the unspiked FCHw-M2 sample, a group of signals was observed between *δ* 1.09 and 1.14, corresponding to an impurity. These signals were used as the internal reference, and their integral was normalized to a value of 100. Consequently, the integrals of the methyl group and aromatic protons were estimated to be 41.36 and 24.67 (Figure 3A). The ^1^H NMR spectrum of the RF-spiked FCHw-M2 sample showed no new signal; however, the integrals of the methyl (114.06) and aromatic protons (68.10) increased substantially (Figure 3B).

**Figure 4:**
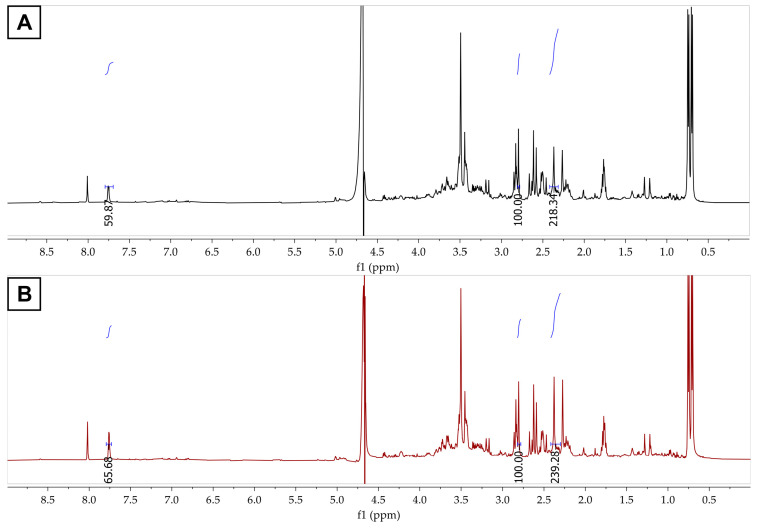
The ^1^H NMR spectra of (**A**) FCHw-M3 and (**B**) FCHw-M3 spiked with riboflavin.

For FCHw-M3, the citrate signal appearing at *δ* 2.80 was considered the internal reference (Figure 4A). The signals of the methyl group (218.34) and aromatic protons (59.87) increased in the spectrum enriched with the RF standard (239.28 and 65.68, respectively) (Figure 4B). These results confirm that the fluorescent compound produced by the *H. wangnamkhiaoensis* yeast strain is RF.

### 3.6. Spectrofluorometric Characterization of the Riboflavin Standard, FCHw-M2, and FCHw-M3

**Figure 5:**
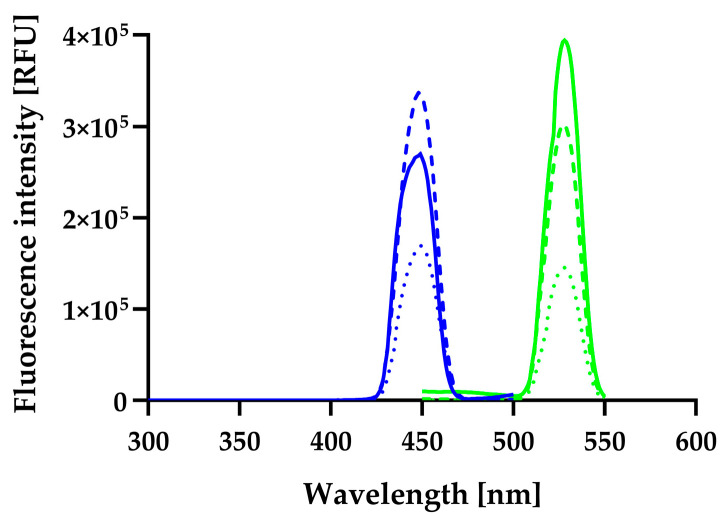
Fluorescence excitation (blue plots) and emission (green plots) spectra of the riboflavin standard (–), FCHw-M2 (---), and FCHw-M3 (···).

The fluorescence excitation and emission spectra of the RF-containing FCHw-M2 and FCHw-M3 and the RF standard were recorded and compared. All fluorescence excitation and emission spectra showed considerable similarities (Figure 5). The maximum emission wavelength (λ_em_max_) of FCHw-M2, FCHw-M3, and the RF standard was 528 nm. The maximum excitation wavelength (λ_ex_max_) of FCHw-M2 was 448 nm, while that for FCHw-M3 and the RF standard was 449 nm. These maximum wavelengths are also in strong agreement with those previously reported for RF of different origins [36,38,42,43,53,86]. These results also support that the yellow fluorescent compound produced by *H. wangnamkhiaoensis* is RF.

From all the above, the HPLC and ^1^H NMR analytical techniques used in our work are powerful and robust analytical tools that provide truly reliable, precise, reproducible, and fast results for the separation and identification of riboflavin. However, their main drawbacks are as follows: (1) Expensive equipment is required, (2) high-quality components are needed, (3) the solvents and/or columns are expensive, (4) regular maintenance and calibration are needed, which add extra cost, and (5) sophisticated software is required for data analysis [87,88].

## 4. Conclusions

A brilliant yellow fluorescent compound excreted into the culture supernatant of *H. wangnamkhiaoensis* was isolated and identified for the first time in the literature. The culture supernatants of *H. wangnamkhiaoensis* were analyzed using three different RP-HPLC-DAD methods, and the desired compound was successfully separated using two out of the three RP-HPLC-DAD methods. The maximum UV–Vis absorption wavelengths (~224, 268, 372, and 448 nm) and the ^1^H NMR signals of methyl groups (*δ* 2.30 and 2.40) and aromatic protons (*δ* 7.79 and 7.77) revealed that the fluorescent compound is riboflavin. The identity of this compound was further confirmed by spiking the ^1^H NMR spectra with riboflavin and spectrofluorometric measurements. This work proposes simple, modern, fast, precise, reliable, sensitive, and reproducible methods for separating and identifying riboflavin, an essential vitamin for overall good health. Furthermore, it also broadens the spectrum of riboflavin-overproducing yeasts, opens new possibilities and perspectives for practical applications of riboflavin production, and triggers new innovative actions. Studies on riboflavin production by batch and single-stage steady-state continuous cultures of the novel *H. wangnamkhiaoensis* yeast strain in a bubble column pneumatic bioreactor are in progress.

## Data Availability

All relevant data are within the paper.
